# LC/MS-based discrimination between plasma and urine metabolomic changes following exposure to ultraviolet radiation by using data modelling

**DOI:** 10.1007/s11306-023-01977-0

**Published:** 2023-02-13

**Authors:** Ali Muhsen Ali, Chris Monaghan, David J. Muggeridge, Chris Easton, David G. Watson

**Affiliations:** 1 College of Medicine, University of Kerbala, Karbala, Iraq; 2 Institute for Clinical Exercise and Health Science, University of theWest of Scotland, Almada Street, Hamilton, Blantyre, ML3 0JB UK; 3 Edinburgh Napier University, Sighthill Court, Edinburgh, EH11 4BN UK; 4 Strathclyde Institute of Pharmacy and Biomedical Sciences, 161, Cathedral Street, Glasgow, G4 0RE Scotland, UK

**Keywords:** LC–MS, Ultraviolet radiation (UV), Urine, Plasma, Metabolomic profiling, Principal components analysis (PCA), Orthogonal partial least squares- discriminant analysis (OPLS-DA)

## Abstract

**Introduction:**

This study sought to compare between metabolomic changes of human urine and plasma to investigate which one can be used as best tool to identify metabolomic profiling and novel biomarkers associated to the potential effects of ultraviolet (UV) radiation.

**Method:**

A pilot study of metabolomic patterns of human plasma and urine samples from four adult healthy individuals at before (S1) and after (S2) exposure (UV) and non-exposure (UC) were carried out by using liquid chromatography-mass spectrometry (LC–MS).

**Results:**

The best results which were obtained by normalizing the metabolites to their mean output underwent to principal components analysis (PCA) and Orthogonal Partial least squares-discriminant analysis (OPLS-DA) to separate pre-from post-of exposure and non-exposure of UV. This separation by data modeling was clear in urine samples unlike plasma samples. In addition to overview of the scores plots, the variance predicted-Q2 (Cum), variance explained-R2X (Cum) and *p*-value of the cross-validated ANOVA score of PCA and OPLS-DA models indicated to this clear separation. Q2 (Cum) and R2X (Cum) values of PCA model for urine samples were 0.908 and 0.982, respectively, and OPLS-DA model values were 1.0 and 0.914, respectively. While these values in plasma samples were Q2 = 0.429 and R2X = 0.660 for PCA model and Q2 = 0.983 and R2X = 0.944 for OPLS-DA model. LC–MS metabolomic analysis showed the changes in numerous metabolic pathways including: amino acid, lipids, peptides, xenobiotics biodegradation, carbohydrates, nucleotides, Co-factors and vitamins which may contribute to the evaluation of the effects associated with UV sunlight exposure.

**Conclusions:**

The results of pilot study indicate that pre and post-exposure UV metabolomics screening of urine samples may be the best tool than plasma samples and a potential approach to predict the metabolomic changes due to UV exposure. Additional future work may shed light on the application of available metabolomic approaches to explore potential predictive markers to determine the impacts of UV sunlight.

**Supplementary Information:**

The online version contains supplementary material available at 10.1007/s11306-023-01977-0.

## Introduction

The direct exposure to sunlight, which is known as a one of main natural sources of UV radiation has beneficial and harmful effects leading to improvement or damage tohuman health. UV radiation can be divided into three regions as follows: UVA radiation with wavelength 315–400 nm, UVB radiation (280–315 nm), and UVC radiation (100–280 nm). UVC radiation which does not reach the earth’s surface because of it is filtering out by the earth’s atmosphere (Lucas et al., 2006). Generally, most of UVA radiation and about 10% of UVB radiation reach and have an important impact on human health (Lucas et al., 2006) and also has an impact on ‘leaf litter decomposition across different biomes’ (Song et al., 2013). Numerous studies have demonstrated clearly the positive effects of UV radiation on human health such as an enhancement of levels of vitamin D (Weller, 2016) and the treatment of: jaundice (Piltingsrud et al., 1976; Salih, 2001), psoriasis (Bataille et al., 2000; Brown et al., 1980), and vitiligo (El-Zawahry et al., 2012). Other studies unveiled a lot of risks and negative effects of UV radiation that include: the acceleration of skin aging, immune system collapse, weakening of eyesight, and cancer (Cejka et al., 2010; Liu et al., 2014; Norval et al., 2008; Surdu et al., 2013). The amount or type of UV exposure are major factors in the development of the many of human illness due to UV radiation exposure such as skin diseases and eye damage (Cejka et al., 2009; Gallagher et al., 2010). In its recent reports, the World Health Organisation indicated that these diseases, generally recognized a being due to overexposure to UV radiation, substantially contribute to mortality rates and cases of permanent disability occurring globally each year. Skin cancers are listed as the leading cause of death 66,000 global deaths per year. In addition, as 20% of the *ca* 15 million cataract cases annually may be caused by sunlight exposure according to estimates by the WHO (WHO, Intersun, The Global UV Project: A Guide & Compendium, 2003).

The metabolic changes showed in plants under stressful exposure to UV light and provide a better understanding of the impacts of UV irradiation on plants (Kaling et al., 2015; Morales et al., 2015; Pandohee et al., 2015; Vidovic et al., 2015; Wargent et al., 2015). Also, scientific researches utilised metabolomic techniques to assess the capacity of UV radiation to induce metabolic modifications in animals. Tessem and his colleagues used (Tessem et al., 2006) ^1^H- nuclear magnetic resonance spectroscopy (^1^H NMR) alone and coupled with high-resolution magic angle spinning (HR-MAS / ^1^H NMR) to describe the relationship between metabolomic changes and the impacts of UV-exposure. They explored that the changes in the metabolic profile after the effects exposure to UV-B radiation had been clearly shown in the rabbit cornea and lens and showed that UV-B had a larger influence than UV-A on aqueous humor composition (Tessem et al., 2005). In another publication by Risa in 2004, the same changes in the metabolic profile of rat lens were identified by using HR-MAS-1H NMR to be further evidence on the effects of the exposure to UV-B radiation (Risa et al., 2004). Mass spectrometry (MS) was used to follow metabolic changes following long-lasting exposure to UV-B radiation in the liver of hairless mouse (Park et al., 2014a). Similar metabolic observations were made in UVB-irradiated mouse skin (Park et al., 2014b). Surprisingly, in spite of many studies have focussed on quantification the effects of UV radiation on metabolite profiling in the non-human subjects such as plants and animals, it is noted that the studies which have been performed in order to examine these effects on human metabolomic profiling were almost non-existent. There was one study by Pearse et al. 1983 and his team which directly linked solar damage and metabolic changes in humans through determining the response of human skin to UV radiation with and without sunscreen protection using. This study demonstrated that the metabolic differences were evident comparing protected and unprotected skin (Pearse & Marks, 1983). There is was recent metabolomic study comparing aqueous humour and lens metabolites from patients with and without cataracts (Yanshole et al., 2019). The application of metabolomic analysis may be a useful tool to enhance understanding of the effects of UV radiation-exposure on humans. One potential benefit of exposure to UV-A radiation is to promote non-enzymatic production of nitric oxide (NO) by the skin (Monaghan et al., 2018). This can have beneficial effects in lowering blood pressure (Liddle et al., 2022). The current aimed to see if metabolomic changes linked to UV exposure to which might indicate effects on NO production.

## Materials and methods

### Chemicals and solvents

HPLC grade Acetonitrile (ACN) was obtained from Fisher Scientific (Loughborough, UK) while Anala R-grade formic acid (98%) was purchased from BDH-Merck (Poole, UK). A Direct-Q 3 Ultrapure Water System (Millipore, Watford, UK) was used to produce HPLC grade water. Ammonium carbonate and methanol (MeOH) were purchased from Sigma-Aldrich (Poole, UK). Authentic stock standards were prepared, as described in previous papers (Zhang et al., 2012, 2014), from standards obtained from Sigma–Aldrich, UK and diluted four times with ACN before LC–MS analysis.

### Subjects and experimental design

This was a pre-post study where measurements were collected before and after the exposure to UVA light. All participants were non-smokers, apparently healthy, and reported no use of medication. Trials were performed in Scotland at 55.78°N latitude between July and December. Each trial was conducted in the morning (before 11 am) after an overnight fast and following the consumption of ~ 500 ml of bottled water upon awakening. the participants were not on any specific diets but samples were taken pre and post exposure in order to eliminate the effect of diet.

**Fig. 1:**
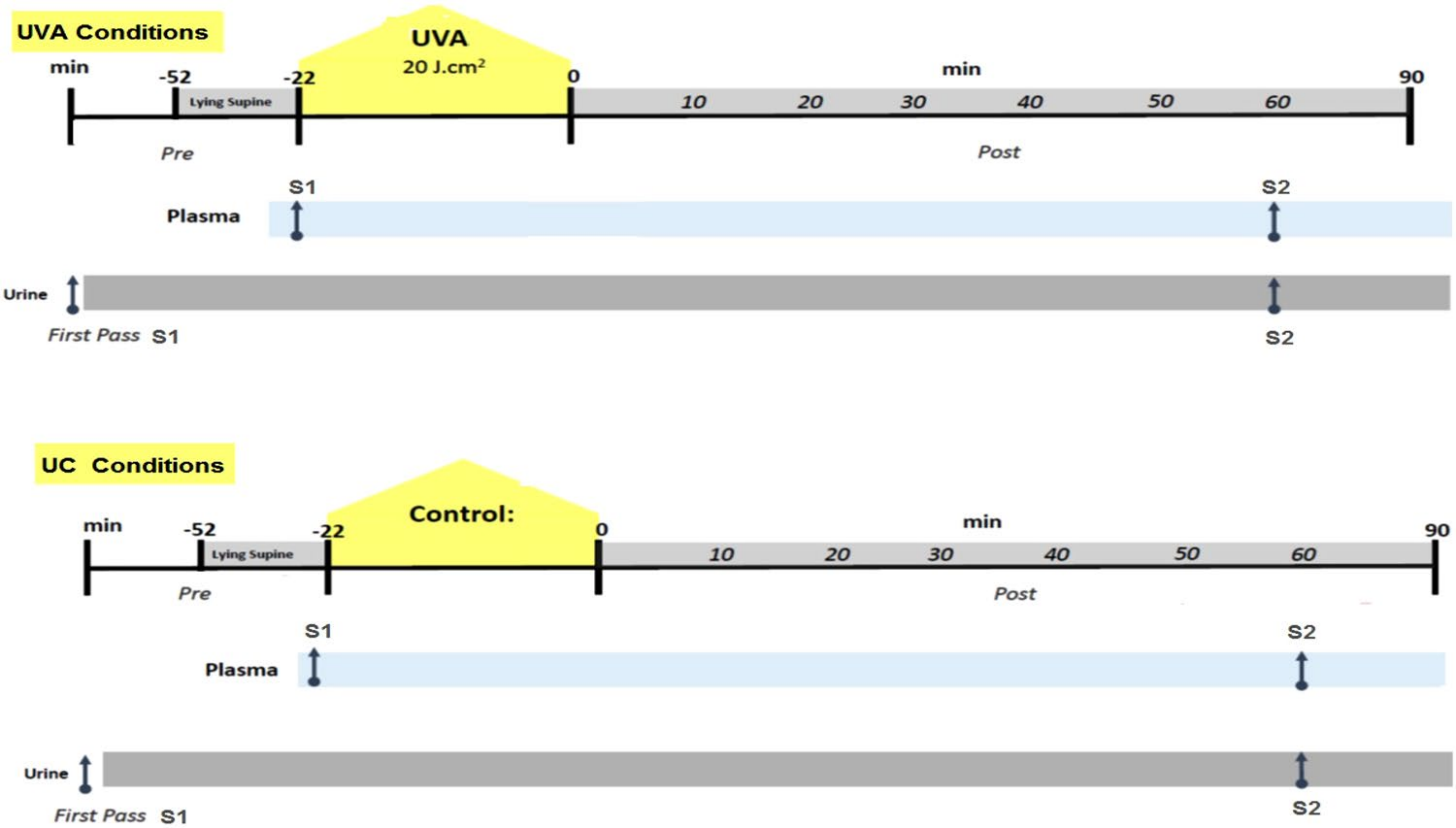
Indicative representation of plasma and urine collection schematic at two consecutive conditions, control conditions, without a dose of UVA light, (UC) and Ultraviolet-A conditions, with a dose of UVA light, (UVA). The first urine sample on each day was the first pass (typically 6 am–8 am)

Human plasma and urine samples were collected from four adult healthy individuals who volunteered and provided written informed consent to participate in the study, which was approved by the ethics committee of the School of Science and Sport, University of the West of Scotland. Each participant visited the laboratory at the School of Sports Science on two consecutive days, control conditions day, without a dose of UVA light, (UC) and Ultraviolet-A conditions day, with a dose of UVA light, (UVA), to provide the plasma and urine samples on two separate time points, first (pre) sample (S1) and second (post) sample (S2) at each day as shown in Fig. 1. Urine fasting samples were collected at first pass to avoid the changes of urinary metabolic profiles due to the different diets (Walmsley et al., 1998**).** In non-UV exposure conditions (UC), plasma and urine samples for each individual were taken at first time point (S1UC) and the second time point sample (S2UC) were after 60 min. All participants in UV-exposure conditions were exposed to a dose of UVA light by 20 J/cm^2^ which is approximately equivalent to 30 min in the sunshine in southern Europe, in summer. The pre- UVA exposure samples (S1UV) and the post-UVA light dose samples (S2UV) were collected.

### HILIC–HRMS analysis conditions

LC–MS-based plasma and urine samples analysis was performed on an Dionex 3000 HPLC (Thermo Fisher Scientific, Hemel Hempstead, UK) combined with an Exactive Orbitrap (Thermo Fisher Scientific) in both positive and negative mode set at 50,000 resolution (controlled by Xcalibur version 2.1.0; Thermo Fisher Scientific, Hemel Hempstead, UK). The mass range (m/z) was scanned at 75–1200 and the capillary temperature was 320 ℃ as well as the flow rates of auxiliary gas and sheath were 17 and 50 arbitrary units, respectively. The separation was carried out by injection 10 μl of each sample solution on a zwitterionic-hydrophilic interaction chromatography column, ZIC-pHILIC column, (150 mm × 4.6 mm; 5 μm from HiChrom, Reading, UK) with mobile phase of (A): 20 mM ammonium carbonate in HPLC grade water (pH 9.2), and (B): HPLC grade acetonitrile (CAN). The samples were kept in a vial tray which was set at 4 °C and a flow rate of mobile phase was 300 μL/min in binary gradient mode which was as follows: 80% of B at 0 min, 20% B at 30 min, 20% B at 36 min, 80% B at 37 min and 80% B 46 min (Zhang et al., 2013, 2016). Preparation of sample in metabolomics analysis mainly depends on type of samples and analysis methods (Issaq et al., 2009). Plasma and urine samples were stored at – 40 ℃ and thawed at room temperature before its preparation. For metabolites urine samples preparation, 200 ul of each urine sample were mixed in clear labelling the Eppendorf tube with 800 ul of ACN. The sample solution was thoroughly mixed by a vortex machine and centrifuged for 15 min with 15,000 rpm at 4 ℃. The clear solution, supernatant, from each samples was transferred to the relevant HPLC vials to be ready for LC–MS analysis. In the preparation of metabolites plasma samples, the protein precipitation by methanol may be the most appropriate way to treatment the protein content in plasma samples in LC–MS approaches to avoid damage to the analytical column and MS capillaries (Bruce et al., 2008). Therefore, the plasma preparation was performed by dilution of 200ul of each plasma sample with 800ul of MeOH/CAN (80/20) then each sample was shaken and centrifuged at 4 ℃ for 15 min/15000 rpm. Finally, the supernatant was transferred onto the correspondingly labelled HPLC vials for analysis.

### LC–MS data processing and statistical analysis

Raw LC–MS files obtained from Xcalibur software were extracted by using m/z Mine 2.14 (Pluskal et al., 2010) in order to metabolite identification by peak extraction and alignment, as previously described (Muhsen Ali et al., 2016; Zhang et al., 2013). House metabolite database was prepared by including data from the Human Metabolome database, Lipid Maps and the Metlin database to be used for search the accurate masses and detect a putative identification of metabolites. The removing of background peaks present in the blank was conducted in MZmine before transferring the data for carrying out univariate analysis. Univariate analysis including normalisation, the area for each metabolite divided to the mean of the peak areas for each metabolite across the samples within each conditions day (UC and UVA), and statistical analysis, paired *t*-test (*p*-value) and fold changes (ratio), were completed in Excel (Microsoft Office 2013). Multivariate analysis were applied by SIMCA-P version 14.0 (Umetrics, Sweden) for data modelling in order to build the models of principal components analysis (PCA), an unsupervised analysis method, and Orthogonal Partial least squares- discriminant analysis (OPLS-DA), a supervised method, (Alonso et al., 2015; Peng et al., 2014). The data were centred, and Pareto scaled for PCA and OPLS-DA were extracted to generate S-plots for visualisation of the components with significant influence in the dataset.

## Results and discussion

In metabolomic profiling studies, choosing the biological fluid samples is the first and important step to monitor and reveal the metabolite changes to provide unique metabolomic fingerprint for understanding the complex transitions of metabolic pathways.

### Pathway and metabolites concentration changes

A pilot study of metabolomic patterns has main role which is used by researchers to choose the appropriate samples for determination metabolomic changes. Changes in metabolites concentration in plasma and urine samples from healthy subjects pre- and post-UV sunlight exposure were established by using LC–MS. Through searching against the data base and matching to a standard by MZmine 2.14, thousands of features in the both samples of plasma and urine in this study were identified to MSI levels 1 or 2 according to their exact mass and retention time (with < 3 ppm deviation). The normalization of peak area for each metabolite across all the time- points that was confirmed by our previous study in order to compare metabolites on the same axis and getting the best modelling for the comparison of data was applied (Muhsen Ali et al., 2016). Univariate analysis of raw data by calculate paired *t*-test and fold changes (ratio) was processed to compare among all time-point samples S1UC, S2UC, S1UV and S2UV in order to determine that the differences are not simply due to the samples being taken on a different time. This comparison among samples found out that the clear metabolomic changes to build multivariate models for our study approaches were between second time point at non-exposure (S2UC) and post-exposure (S2UV). These findings highlighted the identification of metabolites and their pathways in two groups of urine and plasma samples that have been changed according to UV exposure. The data filtering by normalization and the univariate analysis unveiled 121 significant metabolites in urine samples (table S1) and 109 significant metabolites in plasma samples (table S2) from positive and negative ion modes. The selection of these relevant variables that have been significantly changed as a response to these impacts was based on the calculation of the significance value of the variables (*p-value* < 0.05). After the evaluation of selected metabolites, forty-seven of urinary metabolites and forty-four of plasma metabolites which were significantly different by a two-tailed t-tests with threshold 0.05 are identified as important features All of important features were evaluated for their significance applying the false discovery rate statistic (FDR) (Benjamini & Hochberg, 1995) and variable importance in the projection (VIP). VIP was designed in order to assess the contribution of potential variable in an adopted model compared to the rest of variables, the metabolites which have value 1 or higher than this average of VIP will have the greatest contribution in explaining y (Hart & Norval, 2021). Table 1 summarizes the metabolites which have marked difference in its profile by comparison the pre- exposure with post-exposure UV and the non-exposure, at the matched time, in the urine samples. The identified variables which characterized the differences in metabolomic profiling of plasma group at the comparison between the pre and post of the exposure and non-exposure samples are summarized on Table 2. The most of metabolic Pathways including amino acid, lipids, peptide, xenobiotics biodegradation, carbohydrate, nucleotide, Co-factors and vitamins were significantly affected by exposure to UV sunlight. The changes in amino acid metabolism which may attributable to the production of reactive oxygen species or water-loss increase as a result for absorbing of UV were the most important changes. Changes in urocanic acid levels are of interest since it is known to act as a sunlight protectant (Hart & Norval, 2021). Urocanic acid is a metabolite of histidine which is also elevated as it the histidine metabolite methimidazole acetaldehyde. Elevated levels of xanthosine have been found following low level exposure to gamma radiation (Tyburski et al., 2009). Nicotinamide is also important as a UV protecting agent. There were no specific metabolites present which could point to changes in nitric oxide production (Kim & Kirsner, 2010).

**Table 1:** The relevant important metabolites with high impact on the OPLSDA model separating non-exposure from exposure-UV in urine samples based on the critical threshold for a regarding a p-value as being significant is 0.05 and VIP ≥ 1.0. N = negative ion and P

No	Ion mode	m/z	RT	Molecular formula	Name	p-value	Ratio S2UV/S2UC	FDR	VIP
Amino acid metabolism									
1	P	125.07	8.02	C6H8N2O	Methylimidazole acetaldehyde	0.0493	1.525	0.0387	1.00
2	P	156.08	13.70	C6H9N3O2	L-Histidine	0.0162	1.914	0.0315	1.00
3	N	137.04	11.54	C6H6N2O2	Urocanate	0.0121	1.747	0.0315	1.01
4	N	121.00	7.49	C3H6O3S	3-Mercaptolactate	0.0444	1.575	0.0462	1.01
5	N	137.04	7.69	C6H6N2O2	Urocanate	0.0442	1.757	0.0433	1.00
6	P	133.10	23.28	C5H12N2O2	L-Ornithine	0.0063	0.944	0.0315	1.01
7	P	102.05	15.74	C4H7NO2	1-Aminocyclopropane-1-carboxylate	0.0371	0.838	0.0345	1.02
8	N	102.06	12.95	C4H9NO2	4-Aminobutanoate	0.0356	0.751	0.0345	1.01
9	N	174.06	8.94	C10H9NO2	Indole-3-acetate	0.0382	0.551	0.0398	1.02
10	P	156.08	16.33	C6H9N3O2	bacimethrin	0.0167	0.517	0.0315	1.04
11	P	204.12	11.69	C9H18NO4	O-Acetylcarnitine	0.0418	0.561	0.0325	1.05
12	P	88.04	16.40	C3H5NO2	2-Aminoacrylate	0.0203	0.601	0.0315	1.06
13	P	103.04	14.01	C4H6O3	2-Oxobutanoate	0.0096	0.784	0.0315	1.07
14	P	205.12	14.84	C8H16N2O4	N6-Acetyl-N6-hydroxy-L-lysine	0.0428	1.216	0.0387	1.08
15	P	298.10	7.05	C11H15N5O3S	5'-Methylthioadenosine	0.0261	1.548	0.0367	1.10
16	N	152.04	8.72	C7H13NO3	3-Hydroxyanthranilate	0.0119	1.636	0.0315	1.13
17	P	160.10	12.86	C5H9NO3	5-Acetamidopentanoate	0.0497	1.311	0.0497	1.14
18	N	130.05	11.27	C5H6O3	L-Glutamate 5-semialdehyde	0.0039	0.214	0.0312	1.17
19	N	113.02	5.09	C6H12N2O3	2-Hydroxy-2,4-pentadienoate	0.0160	0.294	0.0315	1.17
20	N	159.08	16.01	C7H13NO3	D-Alanyl-D-alanine	0.0283	1.398	0.0325	1.20
Lipids metabolism									
21	N	167.11	6.48	C10H16O2	[PR] (1S,4R)-1-Hydroxy-2-oxolimonene	0.0105	1.553	0.0312	1.02
23	N	363.22	6.06	C21H32O5	Tetrahydrocortisone	0.0079	0.384	0.0312	1.00
24	N	361.20	5.46	C21H30O5	[ST trihydroxy(2:0)] 11beta,17,21-trihydroxypregn-4-ene-3,20-dione	0.0312	0.506	0.0398	1.01
Peptide									
25	P	204.13	15.90	C8H17N3O3	Lys-Gly	0.0212	0.619	0.0345	1.02
26	P	233.15	17.90	C10H20N2O4	Leu-Thr	0.0037	0.575	0.0312	1.04
27	N	489.27	5.93	C25H38N4O6	Ile-Val-Pro-Tyr	0.0417	3.965	0.0345	1.01
28	N	493.23	5.87	C23H34N4O8	Asp-Leu-Phe-Thr	0.0317	0.362	0.0374	1.05
29	P	321.14	12.46	C11H20N4O7	Gln-Ser-Ser	0.0377	0.709	0.0345	0.72
30	P	407.19	8.28	C19H26N4O6	Gln-Pro-Tyr	0.0094	0.487	0.0315	1.02
Cofactors and vitamins									
31	P	123.06	8.08	C6H6N2O	Nicotinamide	0.0120	2.262	0.0315	1.05
32	N	254.09	12.54	C9H13N5O4	2-Amino-4-hydroxy-6-(D-erythro-1,2,3-trihydroxypropyl)-7,8- dihydropteridine	0.0119	2.709	0.0312	1.07
33	P	145.05	11.67	C6H8O4	2,3-Dimethylmaleate	0.0080	0.385	0.0312	1.12
34	N	138.02	13.59	C6H5NO3	6-Hydroxynicotinate	0.0097	0.615	0.0315	1.17
35	Carbohydrate metabolism								
36	N	177.04	14.12	C6H10O6	D-Galactono-1,5-lactone	0.0446	0.744	0.0421	1.09
37	N	177.04	14.81	C6H10O6	D-Glucono-1,5-lactone	0.0383	0.674	0.0433	1.14
38	P	165.08	9.55	C6H12O5	L-Rhamnose	0.0216	0.746	0.0345	1.16
Nucleotide metabolism									
39	N	110.04	10.32	C4H5N3O	Cytosine	0.0158	1.502	0.0315	1.00
40	P	285.08	12.91	C10H12N4O6	Xanthosine	0.0292	1.736	0.0387	1.01
41	P	127.05	9.24	C5H6N2O2	Thymine	0.0131	1.309	0.0315	1.11
Xenobiotics biodegradation and drugs									
42	P	210.09	12.75	C9H11N3O3	4-Acetamido-2-amino-6-nitrotoluene	0.0079	0.380	0.0312	1.01
43	N	195.05	8.15	C6H12O7	D-Gluconic acid	0.0227	1.539	0.0345	1.09
44	N	136.05	29.13	C6H7N3O	Isoniazid	0.0174	1.417	0.0315	1.15
45	N	93.05	7.67	C5H6N2	glutaronitrile	0.0186	1.939	0.0345	1.04
46	P	95.06	13.49	C5H6N2	4-Aminopyridine	0.0416	0.491	0.0315	1.08
Biosynthesis of secondary metabolites									
47	P	167.06	8.84	C5H10O6	L-Arabinonate	0.0308	1.419	0.0345	1.00

**Table 2:** The relevant important metabolites with high impact on the model separating non-exposure from exposure-UV in plasma samples based on the critical threshold for a regarding a P-value as being significant is 0.05 and VIP ≥ 1.0. N = negative ion and P = positive ion

No	Ion mode	m/z	RT	Molecular formula	Name	p-value	Ratio S2UV/S2UC	FDR	VIP
Amino acid metabolism									
1	P	160.10	8.94	C7H13NO3	5-Acetamidopentanoate	0.0220	0.895	0.0357	1.18
2	P	116.07	13.48	C5H9NO2	L-Proline	0.0498	0.875	0.0360	1.13
3	P	190.05	6.64	C10H7NO3	Kynurenate	0.0434	0.878	0.0358	1.09
4	P	164.07	6.64	C6H13NO2S	S-Methyl-L-methionine	0.0399	0.874	0.0357	1.09
5	P	155.05	10.82	C6H6N2O3	Imidazol-5-yl-pyruvate	0.0455	0.862	0.0410	1.09
6	P	298.10	7.07	C11H15N5O3S	5'-Methylthioadenosine	0.0178	0.789	0.0357	1.08
7	P	157.06	11.76	C6H8N2O3	4-Imidazolone-5-propanoate	0.0270	0.911	0.0357	1.07
8	P	118.09	11.88	C5H11NO2	L-Valine	0.0207	0.885	0.0357	1.07
9	N	160.03	15.88	C5H7NO5	N-Formyl-L-aspartate	0.0018	0.457	0.0084	1.06
10	P	154.10	7.76	C7H11N3O	4-(β-Acetylaminoethyl)imidazole	0.0100	0.856	0.0357	1.05
11	P	102.09	14.01	C5H11NO	Betaine aldehyde	0.0314	0.866	0.0368	1.01
12	N	132.03	15.13	C4H7NO4	L-Aspartate	0.0154	1.719	0.0356	1.01
13	N	121.00	10.87	C3H6O3S	3-Mercaptolactate	0.0360	0.863	0.0357	1.00
14	P	222.10	12.46	C8H15NO6	N-Acetyl-D-glucosamine	0.0449	0.843	0.0393	1.00
Lipid metabolism									
15	P	150.11	9.41	C6H15NO3	Triethanolamine	0.0201	0.542	0.0357	1.00
16	P	751.55	3.90	C2H7NO3S	L-α-Phosphatidyl-DL-glycerol	0.0208	0.853	0.0357	1.18
17	P	830.57	4.01	C40H79O10P	1-(9Z,12Z-octadecadienoyl)-2-(4Z,7Z,10Z,13Z,16Z,19Z-docosahexaenoyl)-sn-glycero-3-phosphocholine	0.0419	0.927	0.0393	1.07
18	N	329.18	5.22	C48H80NO8P	Gibberellin A15	0.0365	0.824	0.0394	1.16
19	P	315.16	16.17	C48H84NO7P	[PR] 2,3-Didehydrogibberellin A10	0.0318	0.515	0.0357	1.06
Peptide									
20	P	311.12	14.51	C14H18N2O6	Glu-Tyr	0.0308	0.733	0.0357	1.15
21	N	247.09	15.01	C9H16N2O6	Glu-Thr	0.0055	0.587	0.0259	1.13
22	N	211.08	6.46	C7H16O7	Volemitol	0.0092	0.865	0.0303	1.07
23	P	203.14	14.58	C9H18N2O3	Leu-Ala	0.0085	0.800	0.0259	1.06
24	P	229.15	10.74	C11H20N2O3	Leu-Pro	0.0389	0.830	0.0399	1.05
25	P	177.09	23.16	C6H12N2O4	Ala-Ser	0.0283	0.780	0.0357	1.04
26	N	261.07	15.88	C9H14N2O7	Glu-Asp	0.0179	0.570	0.0356	1.00
27	N	413.17	10.31	C17H26N4O8	Asp-Pro-Pro-Ser	0.0434	1.192	0.0393	1.01
28	P	264.12	15.99	C10H18N4O5	Ala-Ser-Ser	0.0011	0.647	0.0122	1.04
29	P	350.12	9.75	C9H17N3O6	Thr-Asp-Asp	0.0458	0.728	0.0465	1.00
Xenobiotics biodegradation and drugs									
30	N	207.07	5.21	C11H12O4	Benzylsuccinate	0.0127	0.909	0.0356	1.14
31	N	154.05	12.38	C7H9NO3	2-amino-5-methyl-muconate semialdehyde	0.0291	0.896	0.0357	1.13
32	P	476.19	5.50	C24H29NO9	Codeine-6-glucuronide	0.0283	0.897	0.0358	1.12
33	P	138.05	12.38	C7H7NO2	Anthranilate	0.0195	0.833	0.0357	1.09
34	P	168.08	10.67	C7H9N3O2	2,4-Diamino-6-nitrotoluene	0.0181	0.644	0.0356	1.01
35	P	195.08	7.65	C7H6N2O4	4-Aminohippuricacid	0.0366	0.884	0.0376	1.13
36	P	212.10	9.05	C9H10N2O3	Zalcitabine	0.0323	0.821	0.0357	1.10
37	N	293.11	11.85	C9H13N3O3	Aspartame	0.0302	0.724	0.0368	1.09
38	N	291.08	14.97	C14H18N2O5	EDTA	0.0222	0.647	0.0357	1.03
Co-factors and vitamins									
39	P	169.10	5.59	C8H12N2O2	Pyridoxamine	0.0140	0.860	0.0349	1.03
40	N	182.05	5.05	C8H9NO4	4-Pyridoxate	0.0281	0.940	0.0357	1.02
Carbohydrate metabolism									
41	N	145.01	16.03	C5H6O5	2-Oxoglutarate	0.0385	0.654	0.0357	1.06
Nucleotide metabolism									
42	P	115.05	12.85	C4H6N2O2	5,6-Dihydrouracil	0.0246	0.869	0.0356	1.07
Biosynthesis of secondary metabolites									
43	P	186.11	11.74	C9H15NO3	Ecgonine	0.0279	0.906	0.0357	1.08
44	P	241.15	10.61	C12H20N2O3	Slaframine	0.0239	0.820	0.0357	1.04

Multivariate analysis for the modelling of data was conducted by applying two common modeling strategies to reveal patterns in the metabolome differences. Unsupervised PCA and supervised OPLS-DA models were built for biofluid samples of each group for comparing metabolites between pre-exposure vs post-exposure and non-exposure of sunlight. The major diagnostic tools which gives a summary of fit of the PCA and OPLS-DA models and indicates overfitting of the model are the variance explained—R2X(Cum), the variance explained—R2Y(Cum) and the variance predicted-Q2(Cum) (Triba et al., 2015). Table 3 summarise the description of both models for changes in plasma and urine specimen of UC and UV healthy persons, where the godness and overfitting of model depend on a large discrepancy of R2 and Q2 values.

**Table 3:** The statistic description of PCA and OPLSDA models used to describe metabolomic changes in plasma and urine of non-exposure and exposure-UV groups

Model	PCA				OPLS-DA				
Specimen									
	Group	A	R2X(Cum)	Q2(Cum)	Group	R2X(Cum)	R2Y(Cum)	Q2(Cum)	p-value
Plasma	UC:UV	2	0.660	0.429	S1UC:S2UC	0.959	1	0.968	1.0000
					S1UV:S2UV	0.944	1	0.983	0.2446
Urine	UC:UV	7	0.982	0.908	S1UC:S2UC	0.892	0.998	0.996	0.000026
					S1UV:S2UV	0.914	1	1	0.000014

### Metabolic profile and the validation of plasma data models

**Fig. 2:**
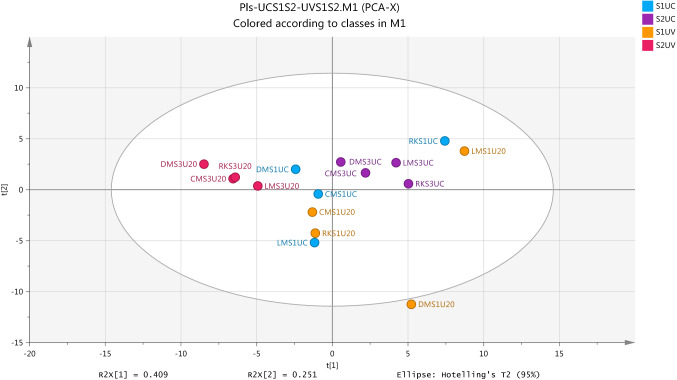
Score Plot for first (S1) and second (S2) samples for each of non-exposure (UC) and exposure-ultraviolet (UV) groups by using PCA (R2X(cum) 0.660, Q2(cum) 0.429, two components) based on the identified metabolites from positive and negative ion modes in Plasma samples

**Fig. 3:**
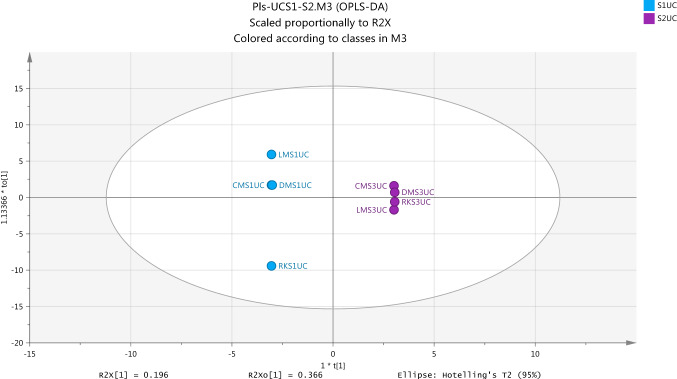
Overview of the observations of OPLS-DA model for separation between two non-exposure samples (S1UC and S2UC) in plasma from healthy subject. R2X(cum) 0.959, R2Y(cum) 1.0, Q2(cum) 0.968

**Fig. 4:**
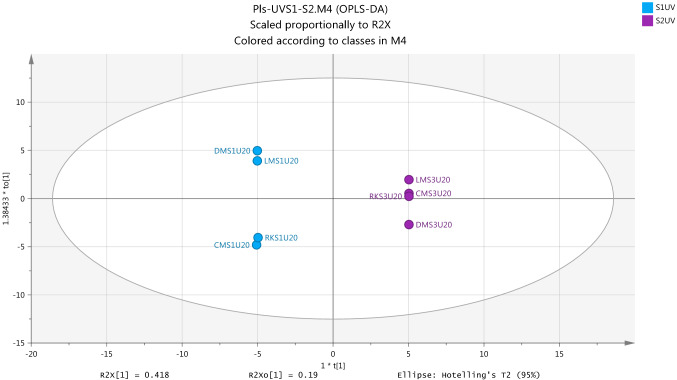
Overview of the observations of OPLS-DA for plasma metabolites with positive and negative ion modes to show the separation between pre- and post-UV exposure samples (S1UV and S2UV). R2X(cum) 0.944, R2Y(cum) 1.0, Q2(cum) 0.983

The data produced from the analysis of plasma samples on the ZICpHILIC column were firstly modeled by unsupervised model in order to get the linear combination formed by reducing single components and the determination of outlier variables. Figure 2 shows the PCA separation of the two groups in the study for the exposure and non-exposure UV following normalisation to individual metabolic output over the four plasma samples collected at two time points for each group. The overview of the observations in Fig. 2 and the values of R2X and Q2 listed in Table 3 indicate that there isno clear PCA separation as a marked difference in metabolite profile among these samples. Moreover, Two OPLS-DA models of the data were made in order to get a clearer picture of the differences between the first and second samples on both UC and UV conditions as show on Figs. 3 and 4, respectively. A glance of observations for OPLS-DA models and the quality of models which was marked by the values of R2X, R2Y and Q2 which are shown in Table 3 predicts that there is clear separation between two samples (S1 and S2) in each group (UC and UV).

In untargeted metabolomic applications, the overview and the internal validation methods such as a variance explained and predicted values are not sufficient to evaluate the quality and goodness of models therefore the validation of data modeling is important step to obtain the reliable results (Trivedi & D., 2012). The validation of OPLS-DA model which was built using SIMCA-P may be carried out by two external methods include cross-validated ANOVA (CV-ANOVA), the returned P-value is indicative of the statistical significance of the investigation, and the permutation test which provides reference distributions of the R2/Q2-value (Worley & Powers, 2013).

**Fig. 5:**
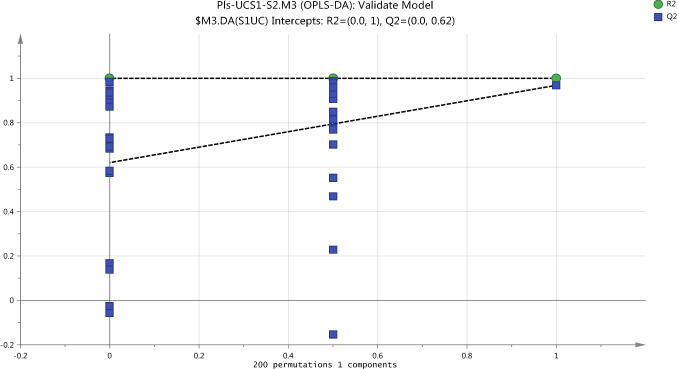
Cross validation of OPLSDA model for the classification of non-exposure group (UCS1-UCS2) in plasma samples by the Permutations test

**Fig. 6:**
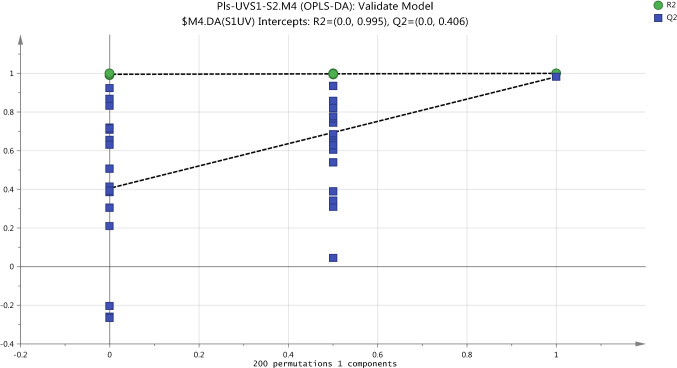
Cross validation of OPLSDA model for the classification of UV-exposure group (UVS1-UVCS2) in plasma samples by the Permutations test

The first external validation methods, CV-ANOVA, of two OPLS-DA models built for plasma data confirm that these models are not fitting and nonsignificant. The returned P-value which was calculated by CV-ANOVA is nonsignificant for non-exposure model, S1/S2-UC, which had score 1.000 and S1/S2-UV exposure model which had score 0.2446. Also, the same results obtained by the second external validation methods or the permutation test provided the evidence on that OPLS-DA models are not fitting. The permutation test gives the reliable assessment for the spurious or credibility of model through the comparison of the goodness of fit (R2 and Q2) of the original model with the goodness of fit of several models based on data. The criteria for model validity is achieved at two conditions include, firstly, all green R2-values should be lower than the original point to the right. Secondly, all blue Q2-values to the left are lower than the original points to the right and the intersection of regression line of the Q2-points with the vertical axis (on the left) occurs at or below zero (Eriksson et al., 2008). The assessment of goodness of the model which was performed by permutation test indicated that S1/S2 non-exposure model is spurious as shown in Fig. 5. Likewise, Fig. 6 shows the validation of the OPLS-DA model to compare between the pre-exposure (S1UV) and post-exposure (S2UV) and indicate that the model is not acceptable.

### Metabolic profile and the validation of urine data models

**Fig. 7:**
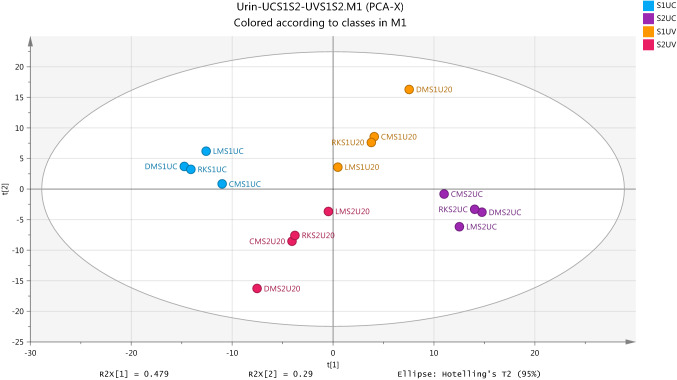
Score Plot for first (S1) and second (S2) samples for each of non-exposure (UC) and exposure-ultraviolet (UV) groups by using PCA (R2X(cum) 0.982, Q2(cum) 0.908, seven components) based on the identified metabolites from positive and negative ion modes in urine samples

The results of urine data modeling showed a largely different profile of metabolites for UV-exposure and non-exposure samples and gave strong indicators on that the modeling was valid. PCA Model was successfully applied to compare between urine groups for the determination of metabolomic profiling approach in response to UV light. Figure 7 shows the PCA score plots of the UC and UV groups to view a clear separation of the subjects according to the profile imbalances of metabolites at pre- and post-exposure and non-exposure to UV radiation. The internal validation method based on the model with the value of R2X (cum) (0.982) and Q2(cum) (0.908) was to be the statistically supported to the application of PCA in the data modelling.

**Fig. 8:**
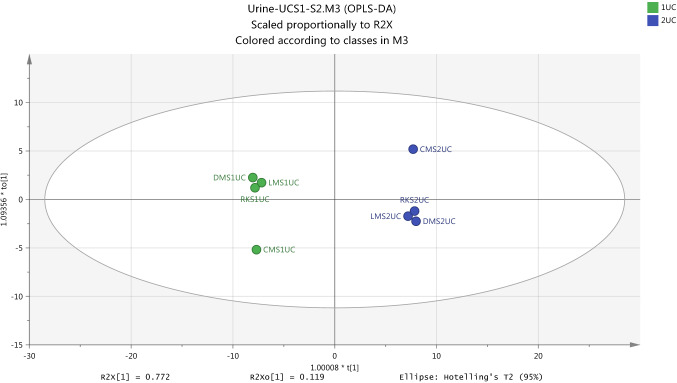
Overview of the observations of OPLS-DA model for separation between two non-exposure samples (S1UC and S2UC) in urine from healthy subject. R2X(cum) 0.892, R2Y(cum) 0.998, Q2(cum) 0.996

Generally, a map of the observations, score plot, which is applied by PCA is used to compress single components and pseudo variables for grouping the original data in the linear form in order to provide the advantage of co-linearity of the data (Liland, 2011). When the linear by score plot is achieved to arrangement data and confiscate dimensionality problems, the understanding of differences between groups by classification model OPLS-DA becomes necessary to separate between samples into each group. The overview of Fig. 8 shows score scatter plot which was generated from OPLS-DA models and indicates that there is clear separation between the two samples (S1 and S2) into non-exposure group (UC). Internally, the validation values of this model which are R2X (cum) 0.892, R2Y(cum) 0.998 and Q2Y(cum) 0.996 were the good evidence on the robustness of the OPLS-DA model in the analysis of a two-class separation of samples.

**Fig. 9:**
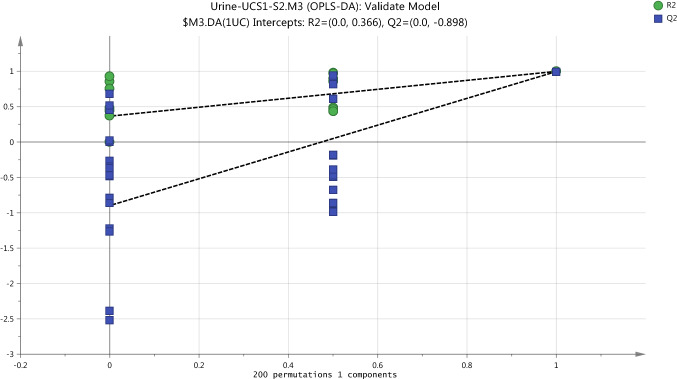
Cross validation of OPLSDA model for the classification of non-exposure group (UCS1-UCS2) in urine samples by the Permutations test

Externally, the validation of the classification model was performed using cross validation-ANOVA and permutation test. CV-ANOVA with P-Value = 0.000026 is significant for model validity in order to use it to inform the selection of samples for statistical comparison. at the permutation test in Fig. 9 shows a strong model since the crossing point of the blue regression line of the Q2-points with the vertical axis is below zero (-0.898), as well as, all green R2-values and blue Q2-values are lower than the original point to the right.

**Fig. 10:**
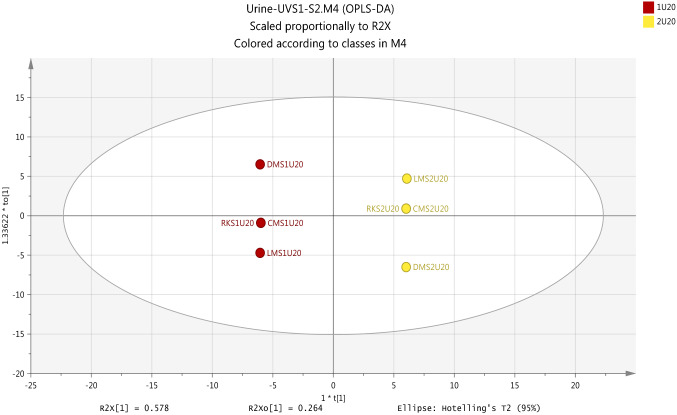
Overview of the observations of OPLS-DA for urine metabolites with positive and negative ion modes to show the separation between pre- and post-UV exposure samples (S1UV and S2UV). R2X(cum) 0.914, R2Y(cum) 1.0, Q2(cum) 1.0

Figure 10 gives shows that an OPLS-DA model was successfully used as a classification model to identify differences in metabolites in urine samples of exposure condition subjects at post-UV exposure (S2) and pre-UV exposure (S1). It is appeared to be a strong metabolic model associated with the effects of exposure to UV based on the significantly value of internal validation tools include R2X(cum) 0.914, R2Y(cum) 1.0 and Q2Y(cum) 1.0.

**Fig. 11:**
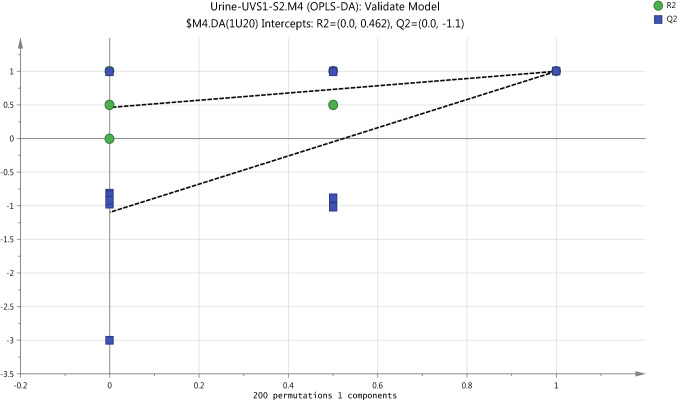
Cross validation of OPLSDA model for the classification of UV-exposure group (UVS1-UVS2) in urine samples by the Permutations test

Cross validation-ANOVA and a permutation test were used to validate the statistical significance of OPLS-DA model which should be valid to determine the metabolome differences in urine samples as a result to exposure to UV. Calculation of P-value (0.000014) which was performed by using the SIMCA-P software and the plot of permutation test as shown in Fig. 11 strongly indicate that the OPLS-DA model is valid.

## Conclusions

The difference in general characteristics between urine and plasma samples such as the ease of collection samples with greater volume than plasma, the high concentrations of particular metabolites in urine and lower urinary protein or lipids contents may be the reasons for the choosing of urine specimens in many of biomedical research. However, these urine properties are not enough to make it to be the best tool in comparison with plasma to provide unique metabolomic fingerprints for understanding the complex changes in metabolic pathways and reveal the metabolites concentration changes. Our pilot study reveal that metabolomic changes of urine samples are more effective in assessing the effects of ultraviolet (UV) radiation compared with plasma samples. The results of pilot study reflected acute changes inmetabolomic patterns, whereas plasma did not, indicating that for pre and post-exposure to UV light screening of urine samples may be the best tool to assessz of metabolomicchanges. Use of PCA and OPLS modelling showed a clear separation for urine metabolites to discriminate between non-exposure and UV-exposure groups provide a possible approach whereas without using this approach the separation was not possible. The current results clearly revealed that the exposure to UV light may affect many of metabolic pathways including amino acid, lipids, peptide, xenobiotics biodegradation, carbohydrate, nucleotides, co-factors and vitamins. In summary, because tno reports on the effects of UV-exposure on human urine metabolome, the study of metabolomic profiling for understanding of the metabolomic changes in human urine following the exposure to UV radiation iswarranted. Although the aim was to see if changes in the metabolic profile indicated effects on markers of nitric oxide metabolism there was no strong indication of such markers in the current study.

## Supplementary Information

Below is the link to the electronic supplementary material.

Supplementary file1 (DOCX 48 KB)

## Data Availability

Raw LC-MS data of human plasma and urine samples and metabolomic data modelling procedure are availableRaw LC-MS data of human plasma and urine samples and metabolomic data modelling procedure are available on request.
