# Supplementary material for: LC/MS-based discrimination between plasma and urine metabolomic changes following exposure to ultraviolet radiation by using data modelling
Source: Metabolomics. 2023 Feb 13;19(2):13. doi: 10.1007/s11306-023-01977-0 (PMC9925544; doi:10.1007/s11306-023-01977-0)
Supplement: Supplementary file 1 — Supplementary file1 (DOCX 48 KB) [file 11306_2023_1977_MOESM1_ESM.docx]

**Table S 1:** The identified metabolites in urine samples after data filtering by normalization and the univariate analysis based on the critical threshold 0.05 for its P-value. N= negative ion and P = positive ion.

| Ion Mode | M/Z | Reten-tion Time | Molecular formula | Name | P-Value S2UC-S2UV | Ratio S2UV/S2UC | P-Value S1UC-S1UV | Ratio S1V/S1UC | P-Value S1UV-S2UV | Ratio S2UV/S1UV |
| --- | --- | --- | --- | --- | --- | --- | --- | --- | --- | --- |
| P | 85.028 | 11.66 | C4H4O2 | 4-Hydroxy-2-butynal | 0.00044 | 0.613 | 0.00044 | 1.508 | 0.0001 | 0.533 |
| P | 126.055 | 11.69 | C6H7NO2 | N-Ethylmaleimide | 0.00191 | 0.501 | 0.00191 | 1.721 | 0.0003 | 0.421 |
| N | 537.234 | 6.60 | C23H34N6O9 | Asn-Lys-Asp-Tyr | 0.00312 | 0.453 | 0.00312 | 3.218 | 0.0317 | 0.572 |
| P | 238.092 | 16.82 | C8H15NO7 | N-Acetyl-D-glucosaminate | 0.00327 | 0.823 | 0.00327 | 1.246 | 0.0899 | 0.919 |
| P | 159.028 | 10.78 | C6H6O5 | [FA methyl,oxo(5:0/2:0)] 2-methylene-4-oxo-pentanedioic acid | 0.00338 | 0.336 | 0.00338 | 3.718 | 0.0005 | 0.369 |
| P | 233.150 | 17.90 | C10H20N2O4 | Leu-Thr | 0.00366 | 0.575 | 0.00366 | 1.879 | 0.0112 | 0.633 |
| N | 130.051 | 11.27 | C5H9NO3 | L-Glutamate 5-semialdehyde | 0.00393 | 0.214 | 0.00393 | 1.334 | 0.0000 | 0.068 |
| P | 283.154 | 6.26 | C15H22O5 | [PR] Artemisin | 0.00435 | 3.325 | 0.00435 | 0.579 | 0.7386 | 1.040 |
| P | 199.096 | 12.49 | C10H14O4 | cis-2,3-Dihydroxy-2,3-dihydro-p-cumate | 0.00503 | 0.588 | 0.00503 | 1.788 | 0.0098 | 0.629 |
| P | 100.076 | 7.91 | C5H9NO | N-Methyl-2-pyrrolidinone | 0.00529 | 1.763 | 0.00529 | 0.639 | 0.0083 | 1.304 |
| P | 143.107 | 6.07 | C8H14O2 | [FA (8:0)] 2Z-octenoic acid | 0.00550 | 0.596 | 0.00550 | 2.147 | 0.1595 | 0.790 |
| P | 133.097 | 23.28 | C5H12N2O2 | L-Ornithine | 0.00631 | 0.944 | 0.00631 | 1.060 | 0.0115 | 0.963 |
| N | 167.046 | 12.62 | C7H8N2O3 | 2,3-Diaminosalicylic acid | 0.00719 | 0.527 | 0.00719 | 2.142 | 0.0225 | 0.595 |
| N | 400.146 | 13.76 | C15H23N5O8 | Asn-Asp-Gly-Pro | 0.00733 | 2.942 | 0.00733 | 0.670 | 0.0018 | 0.746 |
| N | 363.217 | 6.06 | C21H32O5 | Urocortisone | 0.00783 | 0.386 | 0.00783 | 4.267 | 0.0527 | 0.482 |
| P | 210.087 | 12.75 | C9H11N3O3 | 4-Acetamido-2-amino-6-nitrotoluene | 0.00788 | 0.380 | 0.00788 | 3.600 | 0.0335 | 0.442 |
| N | 363.217 | 6.06 | C21H32O5 | Urocortisone | 0.00794 | 0.384 | 0.00794 | 4.260 | 0.0518 | 0.478 |
| P | 145.050 | 11.67 | C6H8O4 | 2,3-Dimethylmaleate | 0.00800 | 0.385 | 0.00800 | 1.859 | 0.0009 | 0.289 |
| P | 241.128 | 10.78 | C10H16N4O3 | Homocarnosine | 0.00845 | 2.930 | 0.00845 | 0.463 | 0.0495 | 1.761 |
| P | 407.192 | 8.28 | C19H26N4O6 | Gln-Pro-Tyr | 0.00941 | 0.487 | 0.00941 | 2.786 | 0.0308 | 0.608 |
| P | 103.039 | 14.01 | C4H6O3 | 2-Oxobutanoate | 0.00960 | 0.784 | 0.00960 | 1.260 | 0.0067 | 0.752 |
| N | 138.020 | 13.59 | C6H5NO3 | 6-Hydroxynicotinate | 0.00967 | 0.615 | 0.00967 | 1.224 | 0.0000 | 0.293 |
| N | 377.146 | 8.58 | C17H22N4O6 | Trp-Ser-Ser | 0.00992 | 1.413 | 0.00992 | 0.825 | 0.0035 | 0.724 |
| N | 180.030 | 8.55 | C8H7NO4 | DIBOA | 0.01034 | 0.411 | 0.01034 | 1.908 | 0.0032 | 0.331 |
| N | 167.108 | 6.48 | C10H16O2 | [PR] (1S,4R)-1-Hydroxy-2-oxolimonene | 0.01047 | 1.553 | 0.01047 | 0.782 | 0.0393 | 0.784 |
| P | 147.048 | 13.98 | C6H10O2S | THTA | 0.01154 | 3.740 | 0.01154 | 0.659 | 0.1115 | 0.708 |
| N | 254.089 | 12.54 | C9H13N5O4 | 2-Amino-4-hydroxy-6-(D-erythro-1,2,3-trihydroxypropyl)-7,8- dihydropteridine | 0.01187 | 2.709 | 0.01187 | 0.288 | 0.0081 | 3.920 |
| N | 152.035 | 8.72 | C7H7NO3 | 3-Hydroxyanthranilate | 0.01190 | 1.636 | 0.01190 | 0.824 | 0.0003 | 0.551 |
| P | 123.055 | 8.08 | C6H6N2O | Nicotinamide | 0.01201 | 2.262 | 0.01201 | 0.512 | 0.0135 | 1.710 |
| P | 160.072 | 16.46 | C5H9O3N3 | formic acid adduct of cytidine | 0.01204 | 0.759 | 0.01204 | 1.257 | 0.0008 | 0.644 |
| N | 137.036 | 11.54 | C6H6N2O2 | Urocanate | 0.01208 | 1.747 | 0.01208 | 0.617 | 0.0303 | 1.449 |
| P | 100.051 | 12.48 | C3H5ON3 | fragment of guanidino acetate | 0.01226 | 0.730 | 0.01226 | 1.368 | 0.0146 | 0.726 |
| P | 199.095 | 12.49 | C10H14O4 | cis-2,3-Dihydroxy-2,3-dihydro-p-cumate | 0.01278 | 0.558 | 0.01278 | 1.806 | 0.0155 | 0.563 |
| P | 280.154 | 8.29 | C15H21NO4 | Metalaxyl | 0.01300 | 0.666 | 0.01300 | 1.406 | 0.0009 | 0.575 |
| P | 127.050 | 9.24 | C5H6N2O2 | Thymine | 0.01314 | 1.309 | 0.01314 | 0.748 | 0.0005 | 1.422 |
| N | 280.105 | 11.54 | C11H15N5O4 | 1-Methyladenosine | 0.01350 | 1.631 | 0.01350 | 0.598 | 0.0106 | 1.736 |
| P | 247.129 | 12.09 | C10H18N2O5 | Glu-Val | 0.01423 | 1.460 | 0.01423 | 0.795 | 0.0432 | 0.821 |
| N | 337.054 | 10.56 | C9H15N4O8P | 1-(5'-Phosphoribosyl)-5-amino-4-imidazolecarboxamide | 0.01461 | 0.185 | 0.01461 | 2.059 | 0.0015 | 0.117 |
| N | 110.036 | 10.32 | C4H5N3O | Cytosine | 0.01581 | 1.502 | 0.01581 | 0.670 | 0.0269 | 1.474 |
| N | 113.024 | 5.09 | C5H6O3 | 2-Hydroxy-2,4-pentadienoate | 0.01597 | 0.294 | 0.01597 | 1.318 | 0.0000 | 0.100 |
| P | 156.077 | 13.70 | C6H9N3O2 | L-Histidine | 0.01622 | 1.914 | 0.01622 | 0.629 | 0.0811 | 1.238 |
| N | 275.078 | 14.18 | C11H16O8 | Ranunculin | 0.01671 | 1.563 | 0.01671 | 0.780 | 0.0029 | 0.785 |
| P | 156.077 | 16.33 | C6H9N3O2 | L-Histidine | 0.01672 | 0.517 | 0.01672 | 2.004 | 0.0347 | 0.537 |
| N | 94.981 | 9.87 | CH4O3S | Methanesulfonic acid | 0.01675 | 1.195 | 0.01675 | 0.845 | 0.0089 | 1.126 |
| P | 541.265 | 6.12 | C27H40O11 | Tetrahydroaldosterone-3-glucuronide | 0.01704 | 0.535 | 0.01704 | 2.098 | 0.0536 | 0.603 |
| P | 275.139 | 5.98 | C15H18N2O3 | indole-3-acetyl-valine | 0.01743 | 6.171 | 0.01743 | 0.634 | 0.1684 | 0.689 |
| N | 136.052 | 29.13 | C6H7N3O | Isoniazid | 0.01743 | 1.417 | 0.01743 | 0.648 | 0.0001 | 1.851 |
| N | 209.057 | 9.48 | C9H10N2O4 | N-carbamoyl-p-hydroxy-D-phenylglycine | 0.01760 | 1.589 | 0.01760 | 0.815 | 0.0019 | 0.614 |
| N | 351.057 | 17.39 | C12H16O12 | 4-(4-Deoxy-alpha-D-gluc-4-enuronosyl)-D-galacturonate | 0.01762 | 1.616 | 0.01762 | 0.683 | 0.0485 | 1.219 |
| N | 167.046 | 8.24 | C7H8N2O3 | 2,3-Diaminosalicylic acid | 0.01773 | 2.088 | 0.01773 | 0.566 | 0.0496 | 1.469 |
| N | 93.046 | 7.67 | C5H6N2 | 4-Aminopyridine | 0.01859 | 1.939 | 0.01859 | 0.509 | 0.0191 | 1.991 |
| P | 88.039 | 16.40 | C3H5NO2 | 2-Aminoacrylate | 0.02033 | 0.601 | 0.02033 | 1.547 | 0.0094 | 0.532 |
| N | 307.115 | 16.33 | C12H24N2O3S2 | S-8-methylthiooctylhydroximoyl-L-cysteine | 0.02058 | 0.712 | 0.02058 | 1.599 | 0.5291 | 0.928 |
| P | 388.160 | 5.40 | C18H21N5O5 | benzyladenine-7-N-glucoside | 0.02078 | 0.000 | 0.02078 | 1.223 | #DIV/0! | 0.000 |
| N | 433.208 | 5.50 | C21H30N4O6 | Ala-Phe-Thr-Pro | 0.02094 | 2.084 | 0.02094 | 0.500 | 0.0344 | 1.919 |
| P | 204.134 | 15.90 | C8H17N3O3 | Lys-Gly | 0.02122 | 0.619 | 0.02122 | 1.708 | 0.0445 | 0.673 |
| P | 165.076 | 9.55 | C6H12O5 | L-Rhamnose | 0.02157 | 0.746 | 0.02157 | 1.175 | 0.0000 | 0.437 |
| N | 185.003 | 7.97 | C8H7O3Cl | 4-Chlorophenoxyacetate | 0.02227 | 1.539 | 0.02227 | 0.771 | 0.1755 | 0.847 |
| N | 195.052 | 8.15 | C6H12O7 | D-Gluconic acid | 0.02270 | 1.539 | 0.02270 | 0.815 | 0.0045 | 0.647 |
| N | 206.051 | 13.66 | C7H13NO4S | S-(2-Hydroxyethyl)-N-acetyl-L-cysteine | 0.02426 | 0.512 | 0.02426 | 1.996 | 0.0297 | 0.523 |
| N | 252.074 | 14.63 | C9H11N5O4 | Neopterin | 0.02431 | 1.469 | 0.02431 | 0.791 | 0.1046 | 0.826 |
| P | 224.113 | 12.53 | C8H17NO6 | N-acetyl -D- glucosaminitol | 0.02515 | 0.798 | 0.02515 | 1.185 | 0.0006 | 0.618 |
| P | 298.097 | 7.05 | C11H15N5O3S | 5'-Methylthioadenosine | 0.02612 | 1.548 | 0.02612 | 0.538 | 0.0044 | 2.425 |
| N | 93.045 | 11.88 | C5H6N2 | 4-Aminopyridine | 0.02645 | 2.771 | 0.02645 | 0.567 | 0.0161 | 1.195 |
| N | 340.072 | 8.58 | C20H11N3O3 | arcyriaflavin C | 0.02652 | 1.735 | 0.02652 | 0.633 | 0.1279 | 1.370 |
| P | 543.280 | 7.63 | C24H34N10O5 | His-Leu-His-His | 0.02653 | 0.568 | 0.02653 | 1.825 | 0.0595 | 0.594 |
| N | 334.126 | 16.57 | C12H21N3O8 | N4-(Acetyl-beta-D-glucosaminyl)asparagine | 0.02723 | 0.600 | 0.02723 | 2.462 | 0.6478 | 0.892 |
| N | 433.208 | 5.50 | C21H30N4O6 | Ala-Phe-Thr-Pro | 0.02733 | 2.021 | 0.02733 | 0.501 | 0.0414 | 1.970 |
| N | 405.177 | 5.44 | C19H26N4O6 | Gln-Pro-Tyr | 0.02734 | 2.167 | 0.02734 | 0.444 | 0.0352 | 2.325 |
| N | 241.012 | 14.72 | C6H11O8P | D-myo-Inositol 1,2-cyclic phosphate | 0.02815 | 1.388 | 0.02815 | 0.787 | 0.7551 | 0.965 |
| N | 159.077 | 16.01 | C6H12N2O3 | D-Alanyl-D-alanine | 0.02833 | 1.398 | 0.02833 | 0.874 | 0.0000 | 0.505 |
| P | 285.083 | 12.91 | C10H12N4O6 | Xanthosine | 0.02922 | 1.736 | 0.02922 | 0.594 | 0.0518 | 1.613 |
| P | 134.081 | 13.90 | C5H11NO3 | 1-deoxyxylonojirimycin | 0.03065 | 1.347 | 0.03065 | 0.844 | 0.0023 | 0.716 |
| P | 167.056 | 8.84 | C5H10O6 | L-Arabinonate | 0.03080 | 1.419 | 0.03080 | 0.798 | 0.0294 | 0.855 |
| N | 361.202 | 5.46 | C21H30O5 | [ST trihydroxy(2:0)] 11beta,17,21-trihydroxypregn-4-ene-3,20-dione | 0.03118 | 0.506 | 0.03118 | 2.124 | 0.0558 | 0.541 |
| N | 239.114 | 10.76 | C10H16N4O3 | Homocarnosine | 0.03171 | 2.352 | 0.03171 | 0.721 | 0.0268 | 0.672 |
| P | 360.128 | 3.97 | C15H21NO9 | Epinephrineglucuronide | 0.03171 | 0.384 | 0.03171 | 1.629 | 0.0028 | 0.241 |
| N | 493.229 | 5.87 | C23H34N4O8 | Asp-Leu-Phe-Thr | 0.03173 | 0.362 | 0.03173 | 1.815 | 0.0065 | 0.255 |
| N | 245.114 | 11.43 | C10H18N2O5 | Glu-Val | 0.03262 | 1.539 | 0.03262 | 0.777 | 0.1006 | 0.820 |
| N | 501.147 | 8.86 | C18H30O16 | alpha-L-Rhamnopyranosyl-(1->2)-beta-D-galactopyranosyl-(1->2)-beta- D-glucuronopyranoside | 0.03289 | 2.416 | 0.03289 | 0.592 | 0.1458 | 1.174 |
| P | 560.307 | 7.06 | C28H41N5O7 | Glu-Leu-Leu-Trp | 0.03302 | 0.586 | 0.03302 | 1.907 | 0.1462 | 0.672 |
| N | 259.093 | 16.20 | C10H16N2O6 | L-alpha-glutamyl-L-hydroxyproline | 0.03377 | 0.798 | 0.03377 | 1.280 | 0.1898 | 0.866 |
| N | 191.056 | 13.41 | C7H12O6 | Quinate | 0.03486 | 1.169 | 0.03486 | 0.909 | 0.0003 | 0.694 |
| P | 161.093 | 12.95 | C6H12N2O3 | D-Alanyl-D-alanine | 0.03499 | 1.493 | 0.03499 | 0.803 | 0.0122 | 0.740 |
| N | 196.075 | 13.91 | C8H11N3O3 | N-Acetyl-L-histidine | 0.03514 | 0.445 | 0.03514 | 2.492 | 0.0018 | 0.481 |
| N | 102.056 | 12.95 | C4H9NO2 | 4-Aminobutanoate | 0.03555 | 0.751 | 0.03555 | 1.390 | 0.0218 | 0.845 |
| N | 624.339 | 8.39 | C29H43N11O5 | Arg-Lys-Trp-His | 0.03584 | 1.760 | 0.03584 | 0.698 | 0.9830 | 1.004 |
| P | 102.055 | 15.74 | C4H7NO2 | 1-Aminocyclopropane-1-carboxylate | 0.03711 | 0.838 | 0.03711 | 1.187 | 0.0284 | 0.811 |
| P | 321.140 | 12.46 | C11H20N4O7 | Gln-Ser-Ser | 0.03766 | 0.709 | 0.03766 | 1.593 | 0.5727 | 0.906 |
| N | 244.059 | 15.77 | C8H11N3O6 | 6-aza-uridine | 0.03819 | 2.156 | 0.03819 | 0.733 | 0.0441 | 0.680 |
| N | 174.056 | 8.94 | C10H9NO2 | Indole-3-acetate | 0.03823 | 0.551 | 0.03823 | 1.736 | 0.0396 | 0.521 |
| N | 177.040 | 14.81 | C6H10O6 | D-Glucono-1,5-lactone | 0.03829 | 0.674 | 0.03829 | 1.208 | 0.0001 | 0.355 |
| N | 146.082 | 13.97 | C6H13NO3 | Fagomine | 0.03906 | 0.514 | 0.03906 | 2.091 | 0.0013 | 0.552 |
| P | 101.071 | 5.17 | C4H8N2O | Gyromitrin | 0.03996 | 1.220 | 0.03996 | 0.857 | 0.0197 | 0.928 |
| P | 299.160 | 5.82 | C15H18N6O | olomoucine | 0.04128 | 2.075 | 0.04128 | 0.431 | 0.0380 | 2.545 |
| P | 95.060 | 13.49 | C5H6N2 | 4-Aminopyridine | 0.04155 | 0.491 | 0.04155 | 1.618 | 0.0071 | 0.368 |
| N | 489.271 | 5.93 | C25H38N4O6 | Ile-Val-Pro-Tyr | 0.04174 | 3.965 | 0.04174 | 0.719 | 0.0307 | 0.522 |
| P | 204.123 | 11.69 | C9H18NO4 | O-Acetylcarnitine | 0.04180 | 0.561 | 0.04180 | 1.574 | 0.0132 | 0.466 |
| N | 211.083 | 15.84 | C7H16O7 | Volemitol | 0.04227 | 1.477 | 0.04227 | 0.716 | 0.2440 | 1.229 |
| P | 110.071 | 16.27 | C5H7N3 | Brunfelsamidine | 0.04255 | 0.771 | 0.04255 | 1.554 | 0.0358 | 1.202 |
| P | 205.118 | 14.84 | C8H16N2O4 | N6-Acetyl-N6-hydroxy-L-lysine | 0.04279 | 1.216 | 0.04279 | 0.891 | 0.0012 | 0.688 |
| N | 147.041 | 16.41 | C4H8N2O4 | O-Carbamoyl-L-serine | 0.04293 | 0.789 | 0.04293 | 1.496 | 0.0592 | 1.238 |
| N | 104.035 | 16.40 | C3H7NO3 | L-Serine | 0.04367 | 0.769 | 0.04367 | 1.437 | 0.9236 | 1.011 |
| P | 182.048 | 12.40 | C5H11NO4S | DL-Methionine sulfone | 0.04382 | 0.689 | 0.04382 | 1.471 | 0.0875 | 0.710 |
| P | 320.181 | 9.09 | C13H25N3O6 | Leu-Thr-Ser | 0.04403 | 1.184 | 0.04403 | 0.811 | 0.0014 | 1.497 |
| N | 137.036 | 7.69 | C6H6N2O2 | Urocanate | 0.04416 | 1.757 | 0.04416 | 0.566 | 0.0650 | 1.778 |
| N | 120.996 | 7.49 | C3H6O3S | 3-Mercaptolactate | 0.04435 | 1.575 | 0.04435 | 0.782 | 0.0651 | 0.765 |
| N | 177.040 | 14.12 | C6H10O6 | D-Glucono-1,5-lactone | 0.04460 | 0.744 | 0.04460 | 1.332 | 0.0048 | 0.726 |
| P | 558.291 | 6.13 | C24H35N11O5 | His-Lys-His-His | 0.04574 | 0.547 | 0.04574 | 1.996 | 0.1290 | 0.603 |
| P | 191.114 | 26.50 | C6H14N4O3 | N-(omega)-Hydroxyarginine | 0.04612 | 2.666 | 0.04612 | 0.334 | 0.0503 | 3.185 |
| N | 541.265 | 7.60 | C24H34N10O5 | His-Leu-His-His | 0.04688 | 0.604 | 0.04688 | 1.684 | 0.0848 | 0.621 |
| P | 163.110 | 14.02 | C6H14N2O3 | N6-Hydroxy-L-lysine | 0.04728 | 0.687 | 0.04728 | 1.383 | 0.0218 | 0.608 |
| N | 155.010 | 10.58 | C5H4N2O4 | Orotate | 0.04830 | 0.722 | 0.04830 | 1.414 | 0.0652 | 0.761 |
| P | 378.139 | 13.26 | C16H19N5O6 | Kinetin-7-N-glucoside | 0.04886 | 1.219 | 0.04886 | 0.790 | 0.0041 | 1.482 |
| N | 244.131 | 5.79 | C10H19N3O4 | Leu-Asn | 0.04922 | 2.949 | 0.04922 | 0.720 | 0.0630 | 0.588 |
| P | 125.071 | 8.02 | C6H8N2O | Methylimidazole acetaldehyde | 0.04930 | 1.525 | 0.04930 | 0.718 | 0.0708 | 1.141 |
| N | 375.130 | 15.30 | C16H24O10 | Loganate | 0.04932 | 2.365 | 0.04932 | 0.733 | 0.0721 | 0.631 |
| N | 539.250 | 6.51 | C27H40O11 | Tetrahydroaldosterone-3-glucuronide | 0.04962 | 0.596 | 0.04962 | 1.716 | 0.0916 | 0.616 |
| P | 160.097 | 12.86 | C7H13NO3 | 5-Acetamidopentanoate | 0.04971 | 1.311 | 0.04971 | 0.877 | 0.0004 | 0.592 |
| P | 118.061 | 16.66 | C3H7N3O2 | Guanidinoacetate | 0.05000 | 0.812 | 0.05004 | 1.209 | 0.0173 | 0.745 |

**Table S 2:** The identified metabolites in Plasma samples after data filtering by normalization and the univariate analysis based on the critical threshold 0.05 for its P-value. N= negative ion and P = positive ion.

| Ion Mode | M/Z | Reten-tion time | Molecular formula | Name | P-Value S2UC-S2UV | Ratio S2UV/S2UC | P-Value S1UC-S1UV | Ratio S1UV/S1UC | P-Value S1UV-S2UV | Ratio S2UV/S1UV |
| --- | --- | --- | --- | --- | --- | --- | --- | --- | --- | --- |
| N | 203.089 | 24.97 | C5H12N6O3 | Dimethylenetriurea | 0.0000 | 0.288 | 0.79112 | 0.848 | 0.2641 | 0.430 |
| P | 269.098 | 12.61 | C20H12O | 2-Hydroxybenzo[a]pyrene | 0.0001 | 0.222 | 0.74211 | 0.788 | 0.3001 | 0.317 |
| P | 237.081 | 26.44 | C9H16O5S | 2-(4'-methylthio)butylmalate | 0.0001 | 0.320 | 0.77380 | 0.847 | 0.2621 | 0.470 |
| P | 299.054 | 18.54 | C16H10O6 | 2',7-Dihydroxy-4',5'-methylenedioxyisoflavone | 0.0002 | 0.172 | 0.90514 | 0.918 | 0.2281 | 0.275 |
| N | 445.128 | 12.21 | C26H22O7 | [Fv] Longicaudatin | 0.0002 | 1.153 | 0.83052 | 1.022 | 0.7704 | 1.025 |
| P | 297.057 | 18.56 | C10H20N2S4 | Disulfiram | 0.0002 | 0.240 | 0.92876 | 0.946 | 0.2398 | 0.364 |
| P | 351.113 | 12.61 | C15H18N4O4S | Biapenem | 0.0004 | 0.225 | 0.69157 | 0.730 | 0.3396 | 0.354 |
| P | 315.187 | 24.97 | C19H26N2S | Pergolide | 0.0010 | 0.268 | 0.90215 | 0.930 | 0.2267 | 0.379 |
| N | 219.036 | 17.79 | C9H8N4OS | thidiazuron | 0.0011 | 0.213 | 0.68278 | 0.757 | 0.2515 | 0.343 |
| P | 264.119 | 15.99 | C9H17N3O6 | Ala-Ser-Ser | 0.0011 | 0.647 | 0.42612 | 1.106 | 0.0382 | 0.672 |
| N | 160.025 | 15.88 | C5H7NO5 | N-Formyl-L-aspartate | 0.0018 | 0.457 | 0.16765 | 1.431 | 0.0439 | 0.487 |
| N | 393.157 | 14.02 | C20H26O8 | Glaucarubolone | 0.0020 | 1.227 | 0.72699 | 1.051 | 0.9779 | 1.002 |
| N | 222.992 | 8.60 | C6H8SO7 | dihydrodihydroxypyranone sulfate | 0.0023 | 0.838 | 0.05138 | 1.136 | 0.0003 | 0.492 |
| N | 356.058 | 15.45 | C16H14F3NO3S | Tolrestat | 0.0030 | 2.707 | 0.99324 | 0.997 | 0.4177 | 1.251 |
| P | 247.092 | 11.11 | C9H14N2O6 | 5-6-Dihydrouridine | 0.0046 | 0.805 | 0.25007 | 1.184 | 0.0720 | 0.741 |
| P | 275.135 | 15.62 | C10H18N4O5 | ala-gly-ala-gly | 0.0049 | 1.519 | 0.20292 | 0.740 | 0.1484 | 1.411 |
| N | 247.093 | 15.01 | C9H16N2O6 | Glu-Thr | 0.0055 | 0.587 | 0.04611 | 1.364 | 0.0019 | 0.526 |
| P | 317.065 | 14.96 | C16H12O7 | Isorhamnetin | 0.0060 | 0.197 | 0.66559 | 0.741 | 0.2729 | 0.373 |
| N | 429.154 | 12.22 | C23H26O8 | Sesartemin | 0.0071 | 1.100 | 0.92654 | 1.009 | 0.9962 | 1.000 |
| P | 190.119 | 15.76 | C7H15N3O3 | L-Homocitrulline | 0.0077 | 0.801 | 0.22694 | 1.088 | 0.0043 | 0.738 |
| P | 332.093 | 14.97 | C20H14NO4 | Sanguinarine | 0.0084 | 0.168 | 0.67326 | 0.735 | 0.2664 | 0.328 |
| P | 203.139 | 14.58 | C9H18N2O3 | Leu-Ala | 0.0085 | 0.800 | 0.61991 | 1.063 | 0.0405 | 0.738 |
| P | 315.067 | 14.96 | C10H19O7PS | Malaoxon | 0.0086 | 0.193 | 0.63775 | 0.718 | 0.2735 | 0.358 |
| N | 211.084 | 6.46 | C7H16O7 | Volemitol | 0.0092 | 0.865 | 0.42216 | 1.065 | 0.0264 | 0.830 |
| P | 198.087 | 9.76 | C8H11N3O3 | N-Acetyl-L-histidine | 0.0092 | 0.729 | 0.67390 | 0.934 | 0.2544 | 0.811 |
| N | 315.051 | 14.97 | C16H12O7 | Isorhamnetin | 0.0096 | 0.210 | 0.66480 | 0.735 | 0.2764 | 0.386 |
| P | 154.097 | 7.76 | C7H11N3O | 4-(beta-Acetylaminoethyl)imidazole | 0.0100 | 0.856 | 0.33243 | 1.093 | 0.0470 | 0.775 |
| N | 151.007 | 8.64 | C4H8SO4 | butenol sulfate | 0.0102 | 0.900 | 0.39365 | 1.069 | 0.0559 | 0.816 |
| N | 313.053 | 14.97 | C10H19O7PS | Malaoxon | 0.0106 | 0.202 | 0.65438 | 0.729 | 0.2718 | 0.369 |
| P | 780.589 | 4.00 | C45H82NO7P | PE(22:4(7Z,10Z,13Z,16Z)/P-18:0) | 0.0124 | 0.435 | 0.71847 | 1.132 | 0.1879 | 0.511 |
| N | 207.066 | 5.21 | C11H12O4 | Benzylsuccinate | 0.0127 | 0.909 | 0.11716 | 1.156 | 0.0118 | 0.673 |
| N | 181.025 | 12.57 | C7H6N2O4 | 2,4-Dinitrotoluene | 0.0129 | 1.049 | 0.33060 | 0.966 | 0.4904 | 1.022 |
| N | 234.984 | 18.64 | C11H6N2Cl2 | Fenpiclonil | 0.0138 | 1.244 | 0.56660 | 1.170 | 0.5248 | 1.137 |
| P | 169.097 | 5.59 | C8H12N2O2 | Pyridoxamine | 0.0140 | 0.860 | 0.17919 | 1.182 | 0.0561 | 0.745 |
| P | 302.138 | 4.64 | C17H19NO4 | 6-O-Methylnorlaudanosoline | 0.0142 | 0.743 | 0.20187 | 1.287 | 0.0761 | 0.645 |
| N | 132.030 | 15.13 | C4H7NO4 | L-Aspartate | 0.0154 | 1.719 | 0.88425 | 0.963 | 0.0559 | 1.359 |
| N | 567.317 | 8.44 | C30H48O10 | Deoxycholicacid3-glucuronide | 0.0160 | 0.793 | 0.41743 | 1.100 | 0.0335 | 0.668 |
| P | 301.142 | 16.05 | C18H20O4 | [Fv Hydroxy,dimethoxy,methy] 2'-Hydroxy-4',6'-dimethoxy-3'-methyldihydrochalcone | 0.0163 | 0.423 | 0.23465 | 1.333 | 0.0386 | 0.459 |
| N | 304.071 | 15.47 | C12H12N7OCl | Phenylamil | 0.0173 | 2.351 | 0.04830 | 0.701 | 0.0729 | 1.441 |
| P | 298.097 | 7.07 | C11H15N5O3S | 5'-Methylthioadenosine | 0.0178 | 0.789 | 0.47857 | 1.089 | 0.0115 | 0.741 |
| N | 261.073 | 15.88 | C9H14N2O7 | Glu-Asp | 0.0179 | 0.570 | 0.09004 | 1.474 | 0.0670 | 0.637 |
| N | 326.125 | 9.74 | C16H17N5O3 | 5,10-methylenetetrahydropteroate | 0.0179 | 0.772 | 0.59724 | 1.122 | 0.1841 | 0.692 |
| N | 349.099 | 12.60 | C15H18N4O4S | Biapenem | 0.0180 | 0.160 | 0.77824 | 0.751 | 0.3568 | 0.259 |
| P | 168.077 | 10.67 | C7H9N3O2 | 2,4-Diamino-6-nitrotoluene | 0.0181 | 0.644 | 0.54653 | 1.114 | 0.0721 | 0.637 |
| P | 236.998 | 18.66 | C11H6N2Cl2 | Fenpiclonil | 0.0182 | 1.274 | 0.54102 | 1.171 | 0.7480 | 1.066 |
| P | 138.055 | 12.38 | C7H7NO2 | Anthranilate | 0.0195 | 0.833 | 0.42732 | 1.099 | 0.0234 | 0.674 |
| P | 175.108 | 16.40 | C7H14N2O3 | N-Acetylornithine | 0.0197 | 0.724 | 0.71834 | 1.046 | 0.0990 | 0.801 |
| P | 150.113 | 9.41 | C6H15NO3 | Triethanolamine | 0.0201 | 0.542 | 0.18934 | 1.793 | 0.1116 | 0.422 |
| N | 124.007 | 15.47 | C2H7NO3S | Taurine | 0.0202 | 1.906 | 0.06188 | 0.763 | 0.0984 | 1.310 |
| P | 118.086 | 11.88 | C5H11NO2 | L-Valine | 0.0207 | 0.885 | 0.38422 | 1.068 | 0.0223 | 0.826 |
| P | 751.548 | 3.90 | C40H79O10P | [PG (17:0/17:0)] 1,2-diheptadecanoyl-sn-glycero-3-phospho-(1'-sn-glycerol) | 0.0208 | 0.853 | 0.16600 | 1.131 | 0.0003 | 0.767 |
| P | 178.086 | 12.49 | C10H11NO2 | 5-Hydroxytryptophol | 0.0211 | 0.805 | 0.13501 | 1.121 | 0.0041 | 0.695 |
| P | 205.084 | 15.94 | C12H12O3 | 3-Butylidene-7-hydroxyphthalide | 0.0218 | 0.717 | 0.13283 | 1.131 | 0.0095 | 0.706 |
| P | 160.097 | 8.94 | C7H13NO3 | 5-Acetamidopentanoate | 0.0220 | 0.895 | 0.07034 | 1.117 | 0.0002 | 0.740 |
| N | 291.083 | 14.97 | C10H16N2O8 | EDTA | 0.0222 | 0.647 | 0.07812 | 1.254 | 0.0354 | 0.735 |
| P | 228.086 | 12.41 | C10H13NO5 | L-Arogenate | 0.0238 | 0.810 | 0.49462 | 1.064 | 0.0071 | 0.695 |
| P | 241.155 | 10.61 | C12H20N2O3 | Slaframine | 0.0239 | 0.820 | 0.76875 | 1.046 | 0.0366 | 0.758 |
| P | 115.050 | 12.85 | C4H6N2O2 | 5,6-Dihydrouracil | 0.0246 | 0.869 | 0.44809 | 1.065 | 0.0286 | 0.790 |
| N | 195.033 | 8.74 | C6H12O5S | 1-thio-&beta;-D-glucose | 0.0255 | 0.843 | 0.40116 | 1.048 | 0.0001 | 0.721 |
| P | 147.022 | 14.37 | C6H7O2Cl | 3-Chloro-cis-1,2-dihydroxycyclohexa-3,5-diene | 0.0267 | 0.822 | 0.79770 | 1.035 | 0.1704 | 0.799 |
| P | 157.061 | 11.76 | C6H8N2O3 | 4-Imidazolone-5-propanoate | 0.0270 | 0.911 | 0.30636 | 1.104 | 0.0308 | 0.796 |
| P | 186.113 | 11.74 | C9H15NO3 | Ecgonine | 0.0279 | 0.906 | 0.42365 | 1.067 | 0.0223 | 0.809 |
| N | 182.046 | 5.05 | C8H9NO4 | 4-Pyridoxate | 0.0281 | 0.940 | 0.15882 | 1.105 | 0.0730 | 0.875 |
| P | 476.192 | 5.50 | C24H29NO9 | Codeine-6-glucuronide | 0.0283 | 0.897 | 0.08445 | 1.164 | 0.0079 | 0.756 |
| N | 222.098 | 14.01 | C8H17NO6 | N-acetyl -D- glucosaminitol | 0.0283 | 1.107 | 0.50807 | 1.057 | 0.4574 | 0.962 |
| P | 177.087 | 23.16 | C6H12N2O4 | Ala-Ser | 0.0283 | 0.780 | 0.30372 | 1.137 | 0.0337 | 0.731 |
| N | 154.051 | 12.38 | C7H9NO3 | 2-amino-5-methyl-muconate semialdehyde | 0.0291 | 0.896 | 0.31558 | 1.054 | 0.0079 | 0.856 |
| N | 337.095 | 12.80 | C16H18O8 | p-Coumaroyl quinic acid | 0.0291 | 0.612 | 0.16390 | 1.154 | 0.0053 | 0.507 |
| N | 293.114 | 11.85 | C14H18N2O5 | Aspartame | 0.0302 | 0.724 | 0.03673 | 1.236 | 0.0087 | 0.660 |
| N | 224.980 | 13.59 | C5H7O8P | [GP (2:0)] 1,2-diacyl-sn-glycero-3-phosphate | 0.0304 | 1.196 | 0.84487 | 1.023 | 0.2682 | 1.089 |
| P | 118.121 | 11.87 | C6H15NO | 2-Methylcholine | 0.0306 | 0.887 | 0.32909 | 1.072 | 0.0200 | 0.816 |
| P | 311.124 | 14.51 | C14H18N2O6 | Glu-Tyr | 0.0308 | 0.733 | 0.20306 | 1.142 | 0.0020 | 0.585 |
| P | 102.091 | 14.01 | C5H11NO | Betaine aldehyde | 0.0314 | 0.866 | 0.29023 | 1.113 | 0.0836 | 0.841 |
| P | 315.158 | 16.17 | C19H22O4 | [PR] 2,3-Didehydrogibberellin A10 | 0.0318 | 0.515 | 0.70428 | 1.071 | 0.0341 | 0.663 |
| P | 223.075 | 4.48 | C7H14N2O4S | L-Cystathionine | 0.0318 | 0.919 | 0.27173 | 1.086 | 0.0687 | 0.866 |
| P | 212.103 | 9.05 | C9H13N3O3 | Zalcitabine | 0.0323 | 0.821 | 0.44922 | 1.062 | 0.0199 | 0.754 |
| N | 326.109 | 15.33 | C12H17N5O6 | Asp-Gly-His | 0.0329 | 1.890 | 0.03939 | 0.590 | 0.0508 | 1.505 |
| P | 230.047 | 13.96 | C12H7NO4 | Resazurin | 0.0338 | 0.709 | 0.75473 | 1.044 | 0.0320 | 0.678 |
| P | 328.139 | 9.75 | C16H17N5O3 | 5,10-methylenetetrahydropteroate | 0.0343 | 0.742 | 0.50834 | 1.161 | 0.1449 | 0.640 |
| P | 380.062 | 15.16 | C10H14N5O9P | N2-hydroxyguanosine 5'-monophosphate | 0.0348 | 0.627 | 0.06220 | 1.422 | 0.0197 | 0.549 |
| N | 120.996 | 10.87 | C3H6O3S | 3-Mercaptolactate | 0.0360 | 0.863 | 0.46913 | 1.084 | 0.0987 | 0.841 |
| P | 188.103 | 14.78 | C7H13N3O3 | 5-guanidino-3-methyl-2-oxo-pentanoate | 0.0361 | 0.884 | 0.25764 | 1.089 | 0.0016 | 0.726 |
| N | 329.176 | 5.22 | C20H26O4 | Gibberellin A15 | 0.0365 | 0.824 | 0.04167 | 1.224 | 0.0009 | 0.609 |
| P | 195.076 | 7.65 | C9H10N2O3 | 4-Aminohippuricacid | 0.0366 | 0.884 | 0.51962 | 1.062 | 0.0098 | 0.719 |
| N | 367.105 | 15.28 | C17H20O9 | O-Feruloylquinate | 0.0373 | 2.510 | 0.05277 | 0.550 | 0.0490 | 2.179 |
| N | 325.125 | 15.60 | C20H19FO3 | 2,2,4-Trimethyl-3-(4-fluorophenyl)-2H-1-benzopyran-7-ol acetate | 0.0374 | 1.738 | 0.09600 | 0.586 | 0.1023 | 1.726 |
| N | 145.014 | 16.03 | C5H6O5 | 2-Oxoglutarate | 0.0385 | 0.654 | 0.36340 | 1.109 | 0.0209 | 0.712 |
| P | 229.155 | 10.74 | C11H20N2O3 | Leu-Pro | 0.0389 | 0.830 | 0.48852 | 1.061 | 0.0202 | 0.770 |
| P | 164.074 | 6.64 | C6H13NO2S | S-Methyl-L-methionine | 0.0399 | 0.874 | 0.57991 | 1.039 | 0.0209 | 0.829 |
| P | 495.131 | 15.01 | C26H22O10 | Salvianolic acid A | 0.0401 | 0.029 | 0.67829 | 0.658 | 0.2818 | 0.098 |
| P | 162.058 | 9.30 | C6H11NO2S | allylcysteine | 0.0407 | 0.855 | 0.45241 | 1.043 | 0.0090 | 0.776 |
| P | 255.046 | 16.52 | C7H14N2O4S2 | L-Djenkolic acid | 0.0414 | 0.241 | 0.59643 | 0.685 | 0.3833 | 0.389 |
| P | 309.129 | 14.58 | C12H24N2O3S2 | S-8-methylthiooctylhydroximoyl-L-cysteine | 0.0419 | 0.746 | 0.46256 | 1.218 | 0.2257 | 0.681 |
| P | 830.570 | 4.01 | C48H80NO8P | [PC (18:2/22:6)] 1-(9Z,12Z-octadecadienoyl)-2-(4Z,7Z,10Z,13Z,16Z,19Z-docosahexaenoyl)-sn-glycero-3-phosphocholine | 0.0419 | 0.927 | 0.26874 | 1.095 | 0.0268 | 0.817 |
| P | 148.076 | 10.63 | C9H9NO | 3-Methyloxindole | 0.0427 | 0.756 | 0.61978 | 1.041 | 0.0152 | 0.703 |
| N | 413.168 | 10.31 | C17H26N4O8 | Asp-Pro-Pro-Ser | 0.0434 | 1.192 | 0.54675 | 0.941 | 0.0692 | 1.198 |
| P | 190.050 | 6.64 | C10H7NO3 | Kynurenate | 0.0434 | 0.878 | 0.06547 | 1.174 | 0.0085 | 0.777 |
| N | 173.996 | 15.67 | C6H6ClNO3 | 2-amino-5-chloromuconate semialdehyde | 0.0444 | 0.600 | 0.01116 | 1.578 | 0.0140 | 0.564 |
| P | 222.097 | 12.46 | C8H15NO6 | N-Acetyl-D-glucosamine | 0.0449 | 0.843 | 0.50025 | 1.116 | 0.1260 | 0.728 |
| P | 205.120 | 11.64 | C8H16N2O4 | N6-Acetyl-N6-hydroxy-L-lysine | 0.0452 | 0.829 | 0.29404 | 1.104 | 0.0153 | 0.702 |
| N | 151.061 | 13.57 | C5H12O5 | Xylitol | 0.0452 | 0.626 | 0.05356 | 1.682 | 0.0607 | 0.598 |
| P | 155.045 | 10.82 | C6H6N2O3 | Imidazol-5-yl-pyruvate | 0.0455 | 0.862 | 0.43046 | 1.056 | 0.0077 | 0.801 |
| P | 350.121 | 9.75 | C12H19N3O9 | Thr-Asp-Asp | 0.0458 | 0.728 | 0.43786 | 1.184 | 0.1044 | 0.607 |
| P | 144.102 | 11.31 | C7H13NO2 | Stachydrine | 0.0472 | 0.868 | 0.26140 | 1.068 | 0.0024 | 0.741 |
| P | 278.123 | 12.52 | C12H23NO2S2 | S-(2-Methylpropanoyl)-dihydrolipoamide | 0.0473 | 0.574 | 0.22153 | 1.638 | 0.0489 | 0.396 |
| P | 389.132 | 11.42 | C12H25N2O10P | Fructoselysine 6-phosphate | 0.0477 | 0.463 | 0.23053 | 1.711 | 0.0878 | 0.415 |
| P | 818.607 | 3.97 | C48H84NO7P | [PC (18:1/22:6)] 1-(1Z-octadecenyl)-2-(4Z,7Z,10Z,13Z,16Z,19Z-docosahexaenoyl)-sn-glycero-3-phosphocholine | 0.0484 | 0.895 | 0.32753 | 1.075 | 0.0138 | 0.880 |
| P | 116.071 | 13.48 | C5H9NO2 | L-Proline | 0.0498 | 0.875 | 0.67446 | 1.027 | 0.0022 | 0.809 |
